# Herbst appliance with skeletal anchorage versus dental anchorage in adolescents with Class II malocclusion: study protocol for a randomised controlled trial

**DOI:** 10.1186/s13063-017-2297-5

**Published:** 2017-11-25

**Authors:** Klaus Barretto dos Santos Lopes Batista, Tatiana Lima, Nathália Palomares, Felipe de Assis Carvalho, Cátia Quintão, José Augusto Mendes Miguel, Yin-Ling Lin, Ting-Li Su, Kevin O’Brien

**Affiliations:** 1 Division of Dentistry, Orthodontics, Universidade do Estado do Rio de Janeiro, Av. 28 de Setembro, 157, Vila Isabel, Rio de Janeiro, CEP: 20551-030 Brasil; 2 Division of Dentistry, Universidade Veiga de Almeida, Rua Ibituruna, 108, Maracanã, Rio de Janeiro, CEP: 20271-020 Brasil; 3 Division of Dentistry, The University of Manchester, Oxford Road, Manchester, M13 9PL United Kingdom; 4 Division of Oral Health Statistics, The University of Manchester, Oxford Road, Manchester, M13 9PL United Kingdom; 5 Division of Dentistry, Orthodontics, The University of Manchester, Oxford Road, Manchester, M13 9PL United Kingdom

**Keywords:** Activator appliances, Dental implants, Orthodontic appliances, Orthodontic anchorage Procedures

## Abstract

**Background:**

The Herbst appliance is an orthodontic appliance that is used for the correction of class II malocclusion with skeletal discrepancies. Research has shown that this is effective. However, a potential harm is excessive protrusion of the lower front teeth. This is associated with gingival recession, loss of tooth support, and root resorption. This trial evaluates a method of reducing this problem.

**Methods/Design:**

The study is a single-center, randomised, assessor-blinded, superiority clinical trial with parallel 1:1 allocation. Male and female young people (10–14 years old) with prominent front teeth (class II, division 1) will be treated in one orthodontic clinic. Group 1 will be treated with the conventional Herbst appliance with dental anchorage and group 2 with the Herbst appliance with indirect skeletal anchorage for 12 months. The primary objective will be to compare the proclination of the lower incisors between the Herbst appliance with dental anchorage and skeletal anchorage. Secondary objectives will be to evaluate the changes occurring between the groups in the mandible, maxilla, lower and upper molars, and in gingival recession and root resorption at the end of the treatment. Additionally, the young patient’s experience using the appliances will be assessed. The primary outcome measure will be the amount of lower incisor proclination at the end of treatment. This will be assessed by cone-beam computed tomography (CBCT) superimposition. Secondary outcome measures will be the changes in the mandible, maxilla, lower and upper molars at the end of treatment assessed by tomography superimposition and the young patient’s experience using the appliances assessed by self-reported questionnaires and semi-structured interviews. The randomisation method will be blocked randomisation, using software to generate a randomised list. The allocation concealment will be done in opaque envelopes numbered from 1 to 40 containing the treatment modality. The randomisation will be implemented by the secretary of the Department of Orthodontics of Rio de Janeiro State University before the beginning of the study. The patients and the orthodontists who will treat the patients cannot be blinded, as they will know the type of appliance used. The technician who will take the CBCT image and the data analyst will be blinded to patients’ group allocation.

**Discussion:**

If this new intervention is effective, the findings can change orthodontic practice and may also be relevant to other forms of treatment in which appliances are fixed to the bones of the jaws. However, if the bone anchoring is not effective, the trial will provide much needed information on the use of this comparatively new development.

**Trial registration:**

ClinicalTrials.gov, protocol ID: NCT0241812. Registered on 26 March 2015.

**Electronic supplementary material:**

The online version of this article (doi:10.1186/s13063-017-2297-5) contains supplementary material, which is available to authorized users.

## Background

Orthodontic treatment is directed at the treatment of malocclusion. This may range from the correction of a few crooked teeth to severe problems associated with craniofacial anomalies. One of the most common orthodontic problems is prominent upper front teeth (class II malocclusion). The prevalence of this condition is high and comprises approximately 50% of orthodontic problems [1, 2]. Prominent teeth are associated with low perception of appearance. This may result in psychosocial problems and teasing [3]. Furthermore, upper front teeth are at risk of being traumatised with subsequent damage or even loss.

Different types of braces have been developed for the treatment of class II malocclusion. These are called functional appliances and they correct the position of the incisor teeth. A popular type of functional appliance is the Herbst appliance. This is fixed to the teeth and has been shown to be very effective. Nevertheless, there are concerns that this appliance can result in harm to the teeth and supporting bone. This is because it moves the lower incisors forwards (proclination). This may result in recession of the gums, resorption of the roots and general concerns about the long-term health of the lower incisors [4–10].

With the intention of solving these problems, we developed a version of the Herbst appliance that is fixed to the bone of the lower jaw and not attached to the teeth (skeletal anchorage); thus, removing the forward directed force on the lower incisors [11–14].

## Methods

### Objectives

#### Primary objective

The primary objective is to compare the effects of the Herbst appliance with either skeletal anchorage or dental anchorage on the position of the lower incisors in the treatment of young people with class II malocclusion.

#### Secondary objectives

1. To evaluate the changes that occurred in the mandible and maxilla, the relationship between the maxilla and mandible, lower molars and upper molars, and in gingival recession and root resorption at the end of the treatment with the Herbst appliance with skeletal and dental anchorage in patients with class II malocclusion
2. To explore young people’s experience of using the two types of Herbst appliance

### Design

**Fig. 1:**
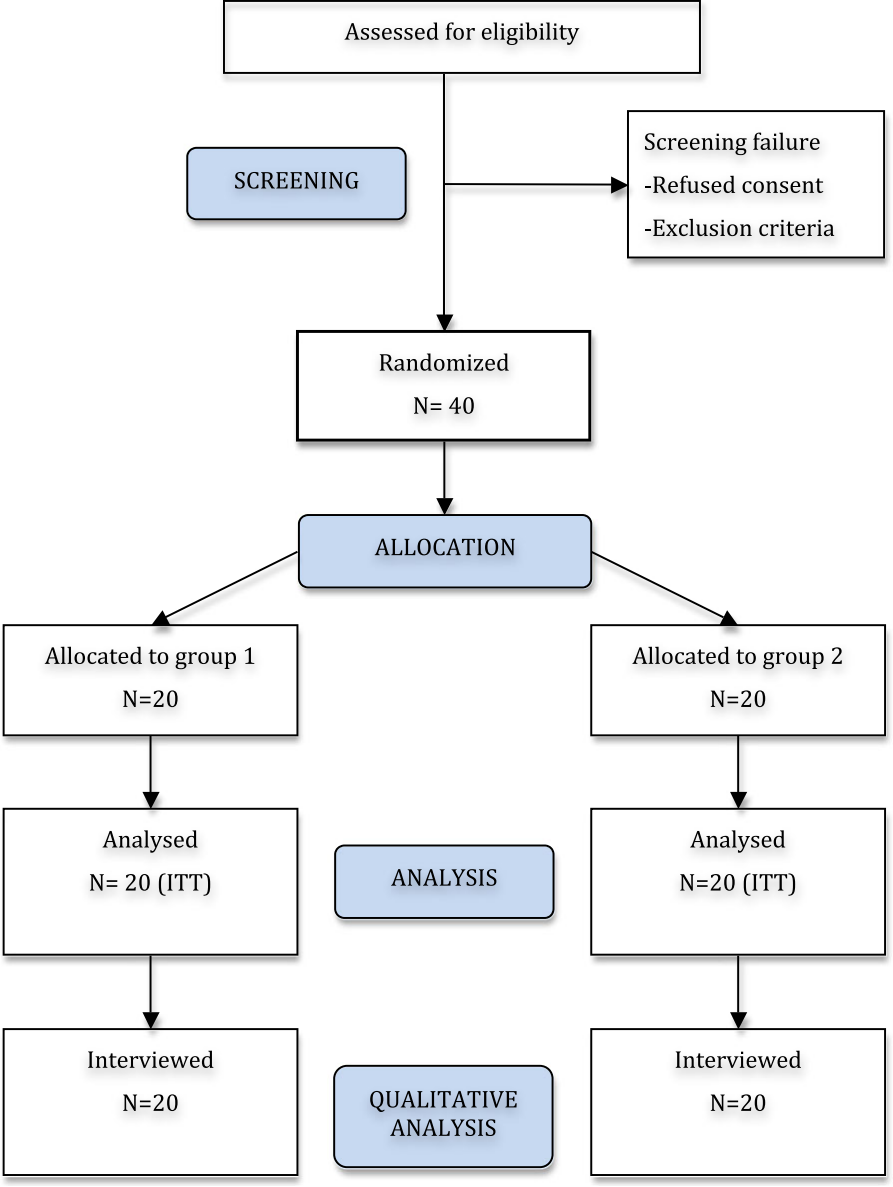
Flow chart with projected numbers of participants throughout the trial. Group 1: Herbst appliance with dental anchorage and group 2: Herbst appliance with skeletal anchorage. *ITT* intention-to-treat

The study is a single-center, randomised, assessor-blinded, superiority clinical trial with parallel 1:1 allocation. It involves children with class II malocclusion. We will randomise 40 children aged from 11 to 14 years old to treatment with a Herbst appliance with either dental or indirect skeletal anchorage. All participants will be followed until the end of the functional (Herbst) appliance phase of treatment. The expected flow of patients through the trial can be seen in Fig. 1. The SPIRIT checklist with the recommended items to address in a clinical trial protocol is available (Additional file 1).

### Setting

#### Participants

Young people aged from 10 to 14 years old with class II malocclusion are eligible to join the trial if they meet the following inclusion criteria:

#### Inclusion criteria

The eligibility criteria for inclusion in the trial will be:

1. Aged from 10 to 14 years old and attending the Universidade do Estado do Rio de Janeiro (UERJ) Orthodontics Clinic at the peak of the pubertal spurt assessed using vertebral analysis
2. Features of a class II, division 1 malocclusion with a convex profile and minimum overjet of 6 mm in permanent dentition, without missing teeth
3. Parental informed consent

For the administering of self-reported questionnaires and semi-structured interviews, the participants will be recruited after they have completed the treatment. The intention will be to speak to all the young people who took part in the trial in the hope of achieving the theoretical data saturation point. A researcher with experience in conducting qualitative research will get in touch with young people via telephone. The researcher will explain the purpose of the interviews to the young people and invite them to take part in the study. The young people will be given at least 48 h to consider their decision and the researcher will call back to confirm the decision.

#### Exclusion criteria

Young people with any of the following are not eligible for inclusion in the trial:

1. Previous orthodontic or orthopaedic treatment with any type of intervention (to avoid confounding factors related to previous treatment)
2. Syndromes, orofacial cleft, or other special needs
3. Missing teeth (to avoid confounding factors related to anchorage loss due to the absence or early extraction of permanent teeth)
4. Poor oral health that precludes orthodontic treatment (presence of caries, active white spots or periodontal diseases)

### Recruitment

The recruitment period will be between August 2015 and August 2018. The strategies for achieving adequate participant enrolment to reach target sample will be:

- Search the waiting file of the UERJ Orthodontics Clinic
- Referral from other public institutions
- Private clinics and public schools

#### Registration and consent

The clinical orthodontist will screen eligible children. If a young person is eligible, the clinician will outline a verbal description of the trial to the young person and their parent/legal guardian. If they are interested in taking part in the trial, the clinician will ask them to give written informed consent. The young people will then give time to read the written informed consent information and ask any questions. The parents/legal guardians who consent to take part will complete the Consent Form, which will also be signed by the young person and the clinician. The parents/legal guardians will be informed that they have the right to withdraw from the trial at any time with the guarantee that their clinical care will not be compromised neither additional costs will be done.

A copy of the written Consent Form will be given to the representatives, and another one will be kept with the investigators who will keep an anonymous screening log of all ineligible and eligible participants who did not consent to take part in the study.

### Locations

The trial will be carried out in the UERJ Orthodontic Clinic, Rio de Janeiro, Brazil. This public university serves a predominantly low-income population located in the Vila Isabel neighbourhood in northern Rio de Janeiro State, Brazil. The estimated population size is 81,858 habitants (IBGE – CENSO 2000). Data will be collected from August 2015 through August 2018.

### Trial intervention

**Fig. 2:**
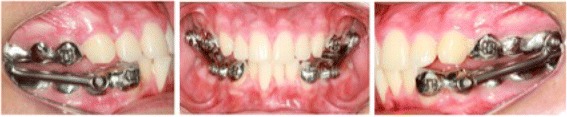
Herbst appliance with dental anchorage

**Fig. 3:**
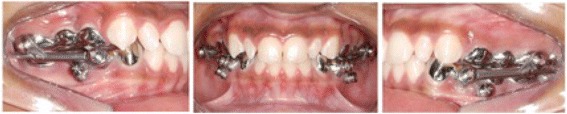
Herbst appliance with skeletal anchorage

Two groups will receive treatment. Group 1 will be treated with the Herbst appliance with dental anchorage for 12 months (Fig. 2). Group 2 will be treated with the Herbst appliance with skeletal anchorage in mini-implants for 12 months (Fig. 3). Only the Herbst phase will be studied to reflect just the changes caused for the orthopaedic appliance.

#### Group 1

The Herbst appliance in group 1 will be the ‘cast splint’ type, made in cobalt-chromium (Herbst I set, Dentaurum, Ispringen, Germany). Group 1 will be anchored from the second molars to the first premolars in the upper arch and from the second molars to cuspids in the lower arch, following the Giessen University protocol [10].

At the first appointment, impressions from the upper and lower teeth will be taken with alginate (Orthoprint, Zhermack, Badia Polesine, Italy). Next, a construction-bite will be taken with wax. Then, the impressions and the registration will be sent to the laboratory for the construction of the Herbst appliance. The Herbst appliance will be placed on the second appointment.

#### Group 2

The Herbst appliance in group 2 will be the same as in group 1. However, its lower part will be anchored to mini-implants using 0.012" stainless-steel ligature wire (Morelli, Sorocaba, São Paulo, Brazil) from the mini-implants to a bracket soldered to the Herbst appliance on the buccal side of the cuspids.

Before placing the mini-implants, a cone-beam computed tomogram (CBCT) will be taken using Classic iCAT tomography (Image Sciences, Hatfield, PA, USA), to select the best site for insertion. We will then insert two self-tapping mini-implants (Neodent, Curitiba, Brazil), 2 mm in diameter × 10 mm in length, with attachments for the Herbst appliance telescopic tubes, between the roots of the cuspids and the first premolars or between the roots of the first and second premolars on each side, on the alveolar ridge through attached gingiva.

A lower rotation handpiece with torque control and a cone-shaped burr 1.3 mm in diameter (Neodent, Curitiba, Brazil) will be used to make a guide from the buccal to the lingual cortical bone in order to achieve bicortical anchorage. After the insertion of the mini-implants, new radiographs will be taken using the parallel technique to check if the mini-implants were inserted in the correct position.

### Analysis

#### Sample size

The primary outcome measure is the difference between the post- and pre-treatment position of the lower incisor edge calculated in millimeters. A 2-mm difference in primary outcome between groups is considered to be clinically significant. This value was obtained based on the clinical judgment of 18 dental professors, senior and junior lecturers, and post-graduate students via interviewing. The standard deviation of such difference was reported in the academic literature to be 1.65 [15]. Allowing for 5% type 1 error, 90% power and equal size allocation among groups, 32 subjects will be required. Considering a 20% attrition rate the total number of recruitment will be 40, with 20 in each group. This calculation was done using online open source software developed by Harvard University [16].

### Randomisation

#### Sequence generation

The randomisation schedule will be prepared and comprise random blocks that are stratified by gender. As a result, the sample will be separated into 10 blocks with four subjects in each.

#### Allocation and concealment mechanism

Allocation sequence will be concealed in sequential opaque envelopes numbered from 1 to 40, with the treatment allocation.

### Implementation

Patients who fulfil the inclusion criteria will be consented before the randomisation. The secretary in the Department of Orthodontics of the Faculty of Dentistry from Rio de Janeiro State University will be responsible for the implementation of the randomisation (generation and storage of the randomised list, allocation, concealment, and treatment assignment). The subjects will write their names on the numbered envelopes and will open them to know the treatment for which they were selected. After that, the envelopes will be closed with the type of treatment selected for storage of the information.

### Blinding

Due to the nature of the treatment, the patients cannot be blinded to the allocation group. Similarly, the orthodontists who will treat the patients cannot be blinded, as they will know the type of appliance used. However, the technician who will take and analyse the CBCT image will be blinded to the allocation cone because the images will not include the appliance. The data analyst will be blinded to group allocation.

### Data collection

#### Baseline

We will complete an eligibility Case Report Form (CRF). A Participant Registration Form will also be completed by the clinician. The clinician will also complete the CRF. This will include data on clinical examinations, clinical records, intra- and extra-oral photographs, study models, and CBCT. At the beginning of the treatment with the Herbst appliance, all patients will be photographed with the appliance.

#### In-treatment data

The patients will be photographed after any new mandibular advancement has been done in the appliance during the treatment.

#### End of treatment

At the end of the treatment with the Herbst appliance, the following data will be collected; intra- and extra-oral photographs, study models, and CBCT.

We will also carry out a qualitative analysis of the interventions. This will be done at the end of treatment. Young people will be given the choice to be interviewed on a face-to-face basis at their clinical appointment or via telephone. A semi-structured interview approach is selected due to its flexible and interactive nature. This will be able to encourage the young people to share their experience freely guided by a series of questions. A topic guide will be used in the interviews to ensure that all topics of interests are covered but it will not be followed strictly. The topic guide will evolve as the data collection progresses to suit the emerging themes. Interviews will take a conversational style to empower participants to share their experience without leading questions.

**Fig. 4:**
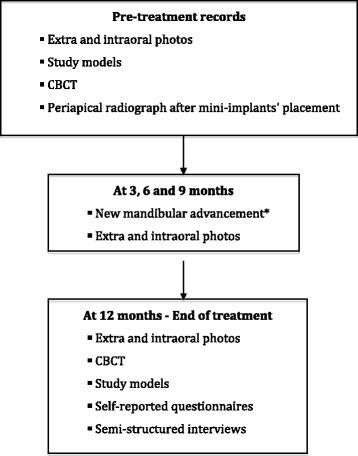
Key steps flow chart. Showing the pre-treatment, treatment and end-of-treatment records collected. *The mandibular advancement will be done just when necessary

The key steps for the records can be seen in the flow chart for the trial (Fig. 4). This will be used as a reference for the involved in the trial.

## Outcome measures

### Primary outcome measure

The primary outcome measure will be the change in the position of the lower incisors. This will be assessed by using CBCT that will be performed at the beginning of the treatment with the Herbst appliance (T1) and at the end of the treatment with the Herbst appliance (T2) using the Classic iCAT tomography (Image Sciences, Hatfield, PA, USA).

All the examinations will be obtained with the patient seated with the mandible in a centric relationship. The field of view (FOV) will be a cylinder with a height of 17 cm per 23-cm diameter. The voxels dimensions will be 0.4 × 0.4 × 0.4 mm.

**Fig. 5:**
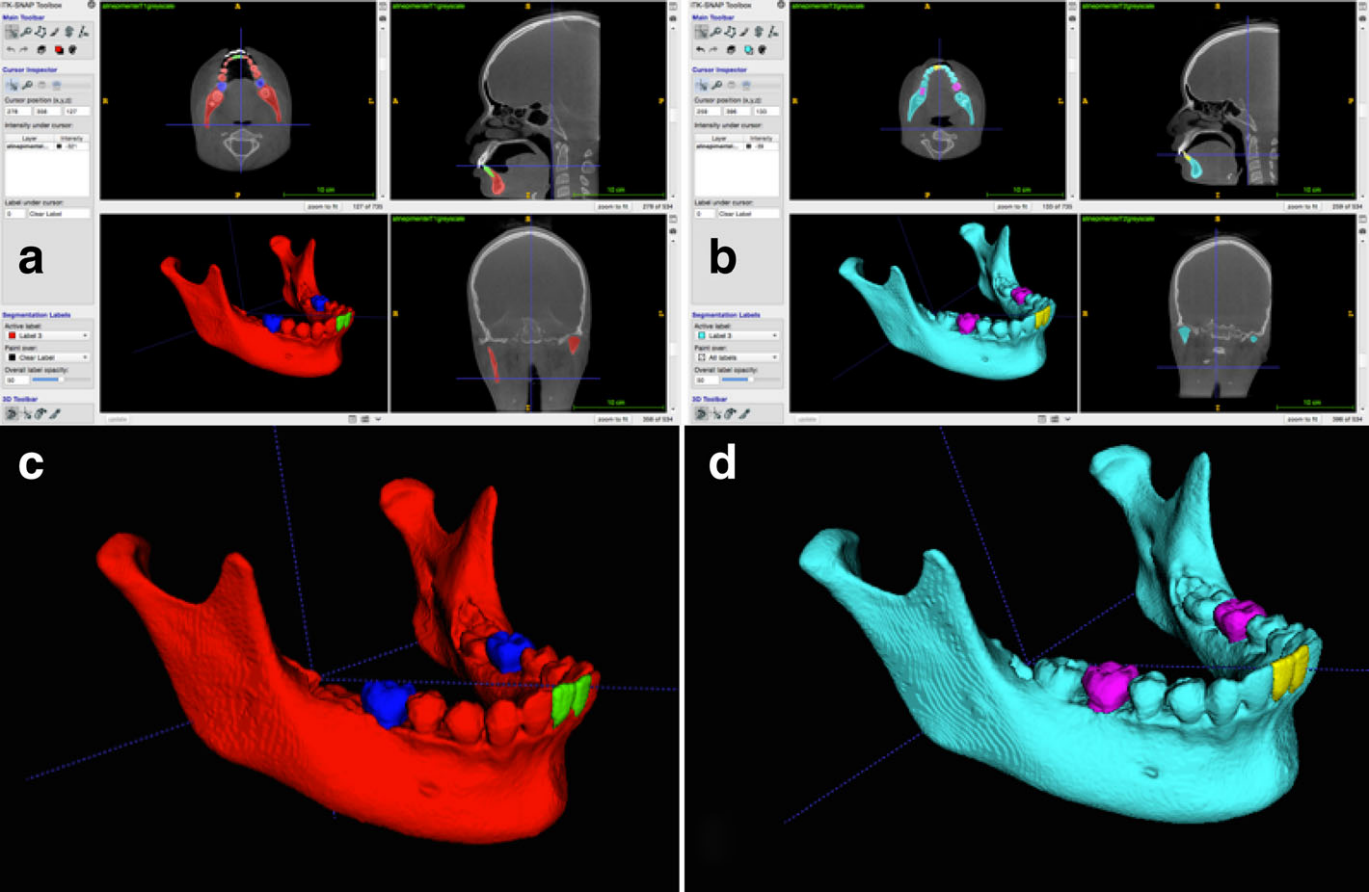
View from the computer screen with the 3D virtual models done using the software ITK-SNAP 3.4. **a** Axial, sagittal and coronal slices and 3D model in T1. **b** Axial, sagittal and coronal slices and 3D model in T2. **c** Approximated view from T1. **d** Approximated view from T2

The tomography image archives will be exported in the Digital Imaging and Communication in Medicine (DICOM) format and converted to a GIPL format (Guys Image Processing Lab) using the open source software ITK-SNAP 3.4 (Fig. 5).

**Fig. 6:**
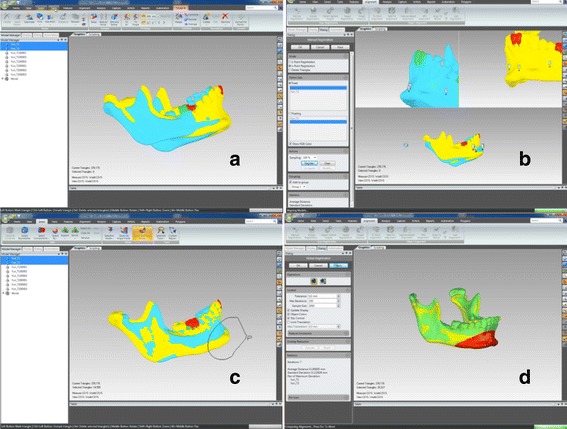
Superimposition of the mandible. **a** Models in T1 and T2 without registration. **b** Registry of the three points in the mandible. **c** Selection tie tool on symphysis. **d** Regional superimposition using the command Global Registration

Single files will be created in .STL format (StereoLithography) using ITK-SNAP 3.4 software and then in VRML format (Virtual Reality Modeling Language) using a .WRL extension (Virtual Reality World). Next, an automatic superimposition from these models will be made using the software Geomagic Qualify 2013 (Geomagic U.S. Corp, Research Triangle Park, NC, USA). Finally, the superimposition will be obtained through the command Global Registration, which perform the best adaptation of the models (Fig. 6). The entire process of models’ surface registration will be based on the calculation of the smaller distance between the surface’s dots in the time points evaluated, aiming the best adaptation between them automatically (*best-fit*).

**Fig. 7:**
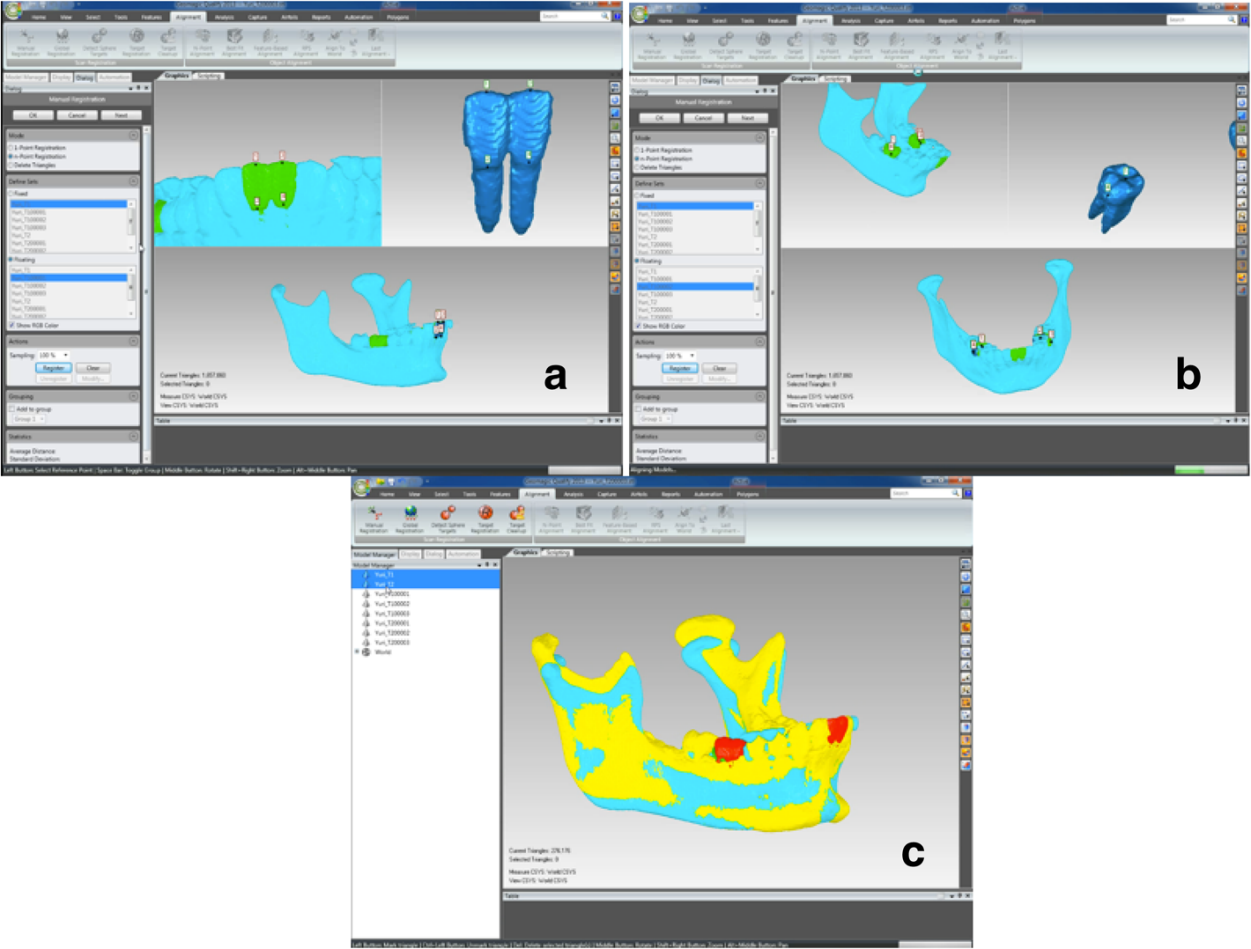
Dental and mandible superimposition. **a** Incisor surfaces to be superimposed. **b** Molars surfaces to be superimposed. **c** Mandibles from T1 and T2 superimposed showing central incisors and molars

For the dental evaluation, the models of the central lower incisors will be imported and superimposed with its respective mandibles using the anterior contour of the chin as a reference (Fig. 7) [17]. After the superimposition, the virtual models will be exported to the .STL format.

**Fig. 8:**
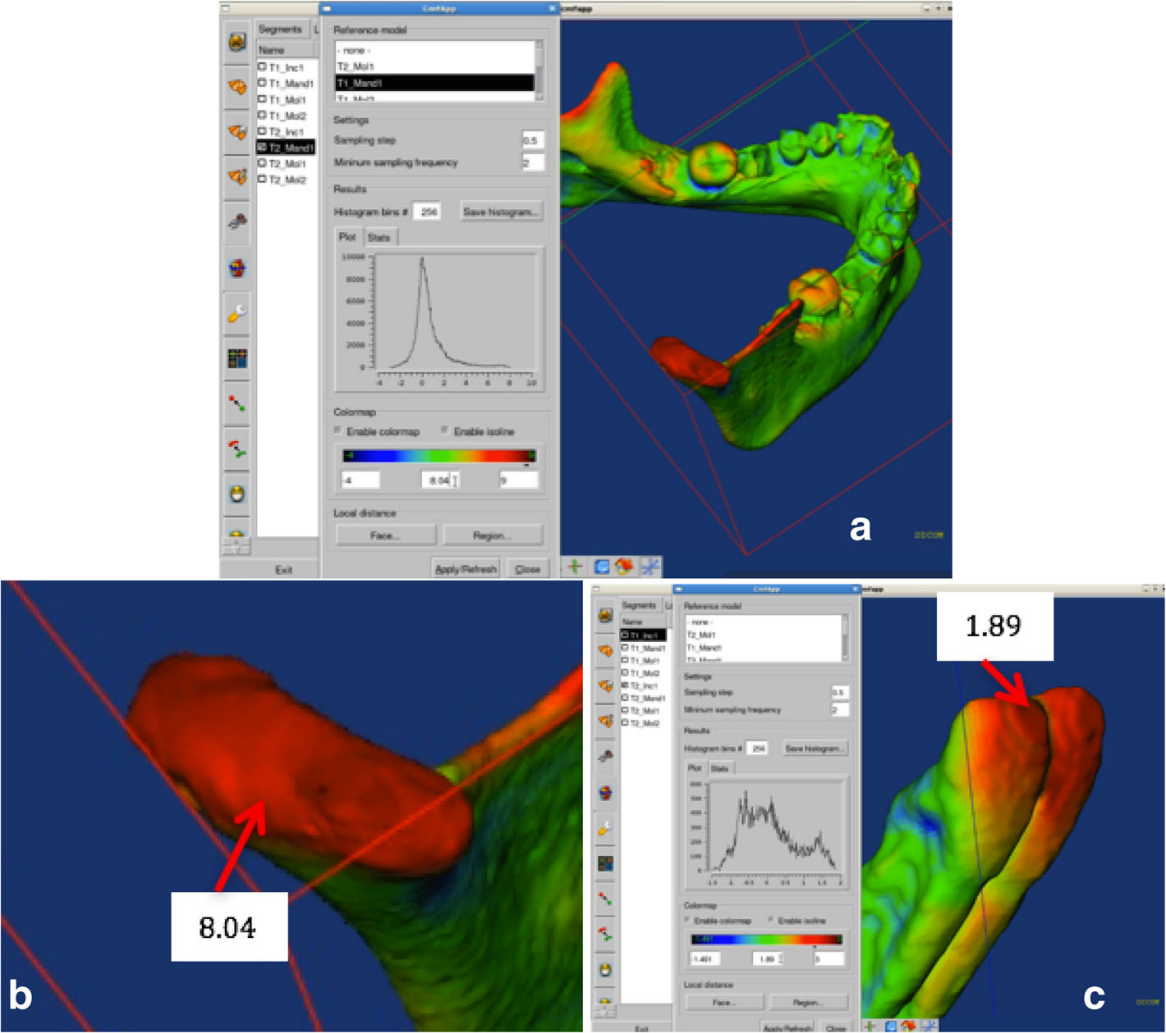
Isoline tool. **a** Growth in the right condyle. **b** Right condyle in a approximated view showing a growth of 8.04 mm in an upper direction. **c** Lower central incisors showing a displacement of 1.89 mm buccally

Next, the software STL2Meta, and the software MetaToIV will be utilised to convert these files to META format and next to .IV format (SGI Open Inventor). The files in. IV format will be imported using the software CMF Application (Maurice Müller Institute, Bern, Switzerland) to quantify mandibular and dental changes between the time points. The contour line tool (Isoline) will be used to identify the most displacement occurred in a region of interest (Fig. 8).

### Secondary outcomes measure

1. To evaluate the bony changes occurring in the mandible, maxilla, the relationship between maxilla and mandible, lower molars and upper molars at the end of the treatment, the same steps used to access the primary outcome measurements will be followed. For the skeletal changes of the mandible, the maxilla and for the relationship between them, the virtual models will be superimposed in the cranial base using the software Geomatic Qualify 2013 (Fig. 9).

**Fig. 9:**
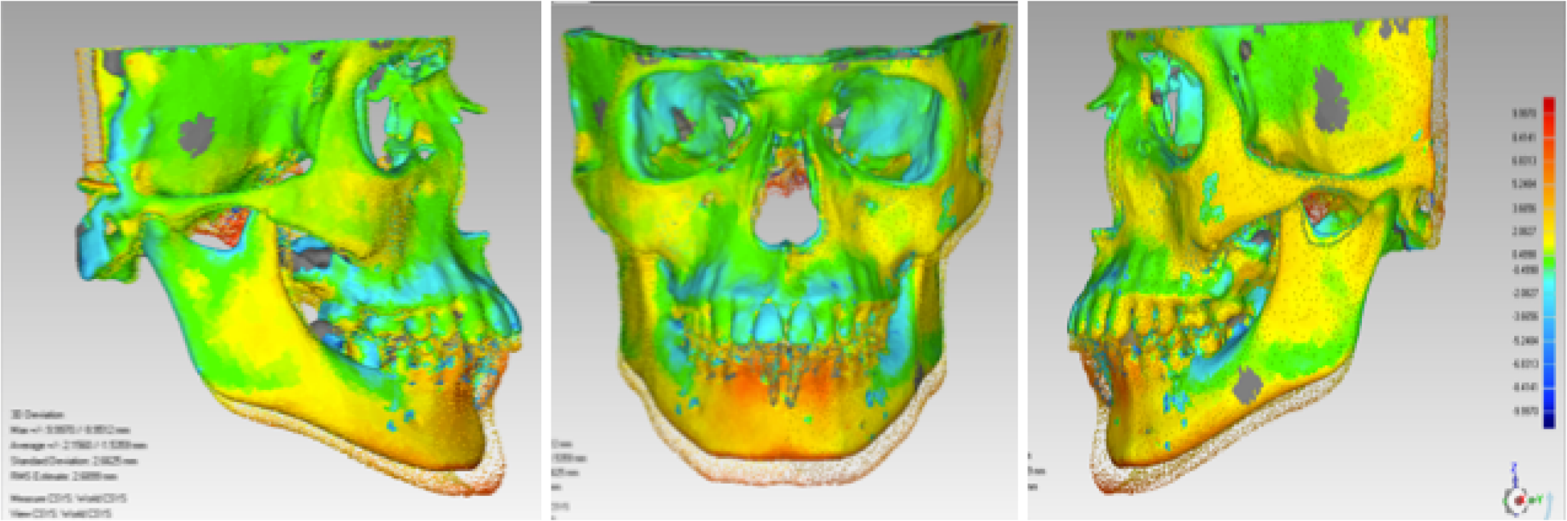
Print screen of the mandible and the maxilla’s superimposition on the cranial base using the software Geomatic Qualify 2013
2. To evaluate gingival recession in the lower incisor area, comparison between dental casts and photographs from the beginning and the end of the Herbst appliance phase will be done. The presence or absence of gingival recession for each lower incisor will be noted for each lower incisor for each time point. In the presence of recession, a ‘yes’ will be marked. The photographs will be used to confirm [18]
3. To evaluate root resorption, comparison between CBCT from the beginning and the end of the Herbst phase will be used for each lower incisor
4. To explore young people’s experience of using the two types of Herbst appliance, the following methods will be used:
  4.1. Self-reported questionnaire about patient discomfort
  4.2. Semi-structured interviews

#### Administrating self-reported questionnaire about patient discomfort

A 5-point Likert scale will be used to access patient discomfort. Measurements of discomfort will be made at the end of treatment, where a score of 1 indicates ‘no pain’ and a score of 5 ‘severe pain’. A mean score will be calculated for each group and compared to each other using paired Student’s *t* test.

#### Semi-structured interviews

Semi-structured interviews will be administered to explore young people’s experience of wearing the two types of Herbst appliance. This is to gain insights into users’ views about the treatments and the impacts, if any, that the appliance had on their everyday life.

### Statistical methods

Patients’ background characteristics as age, gender, as well as their dental and skeletal pre- and post-treatment measures will be summarised using descriptive statistics, such as means, standard deviations, median, and ranges.

Student’s *t* test will be used to compare the changes in lower incisor position between the dental anchorage group and indirect skeletal anchorage group. The results from multivariate analysis, which consider potential confounders, will be reported. The normality of the data will be judged by the Kolmogorov-Smirnov test. Non-parametric equivalent will be applied if any model assumption is not met. The significance level will be set at ≤ 5% and all analysis will be conducted using SPSS statistical software package (version 12.0, Chicago, IL, USA).

Student’s *t* test will be used to assess dental and skeletal differences between the Herbst with dental anchorage and the Herbst with indirect skeletal anchorage. Intra-examiner correlation coefficients (ICC) will be used to evaluate the reliability of repeated measures. A one-sample test will be performed on duplicate measurements to test for systematic errors. The significance level will be set at ≤ 5%.

### Qualitative data analysis

All the interviews will be voice-recorded and transcribed verbatim. Transcripts will be imported to qualitative analysis software (NVivo) for data management and analysis. Framework analysis will be used to explore different cross-sectional descriptive data to capture different aspects of young people’s experience [19]. The researcher will read and re-read the data to familiarise herself with the initial emerging ideas. Codes then will be generated in a systematic fashion across the entire dataset and data will be assigned to the themes formed by the coding exercise. The researcher will then summarise and synthesise the coded data and refine the initial themes whilst identifying the associations and patterns, if any, between the themes. The researcher will then provide interpretation for the refined themes and seek for wider applications of the themes [19].

### Intention-to-treat analysis and imputation

We will carry out an intention-to-treat analysis. As a result, all patients randomised to participate in the trial will be evaluated in the final analysis even if they did not finish the trial or had the data uncompleted. Any missing data will be imputed using multiple imputation procedures. Missing data will be then replaced with a probable value based on other available variables in the data [20].

### Patient’s timeline

**Fig. 10:**
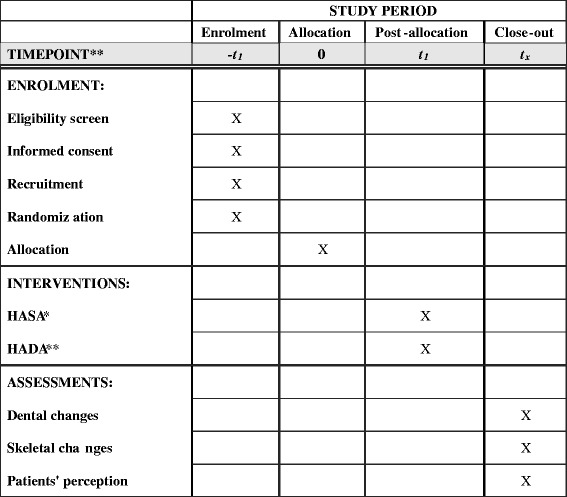
Patient’s timeline. *Herbst appliance with skeletal anchorage. **Herbst appliance with dental anchorage

The schedule of enrolment, interventions, and assessments can be seen in Fig. 10.

## Discussion

Orthopedic appliances have been used for many years to provide treatment for complex orthodontic problems. The Herbst appliance is a fixed functional appliance, which is becoming widely used worldwide. One of the side effects observed after its use is a vestibular inclination of the lower incisors. If this is not controlled the probability of relapse and other harms may be high.

This trial will only be the second trial of the Herbst appliance and the first to evaluate the potential of skeletal anchorage. As a result, the trial will generate much needed evidence for this rather complex form of orthodontic treatment. It will also serve as a model for other trials of orthodontic treatment techniques.

If we find that this new intervention is effective, the findings will change orthodontic practice and may also be relevant to other forms of treatment in which appliances are fixed to the bones of the jaws. However, if the bone anchoring is not effective, the trial will provide much needed information on the use of this comparatively new development. Finally, this will be the first trial that uses CBCT technology to measure tooth and skeletal bone change. This would be an important development and be a model for future research that uses this new method of image capture and analysis.

### Trial status

The trial has been recruiting since August 2015, with an expectation to finish the recruitment in August 2018.

### Safety reporting

#### Adverse events

In the case of adverse events, we will consider two types of adverse event:

1. Non-serious adverse event: this will be any kind of adverse event resulting from the treatment. This will include minor pain, injuries, bruises or wounds to the oral tissues caused by some parts of the appliance, loosening or breakage of the appliance and failure of the mini-implants. In this case, the adverse event will be recorded in the patients’ notes. If any problem occurs to the appliance or mini-implant, it will be fixed and the participant will continue the treatment by the investigators
2. Serious adverse event: this will be any kind of adverse event resulting from the appliances’ use which causes harms to the patient. This will include the swallowing of parts of the appliance, high levels of dental pain or temporomandibular joint, extensive problematic injuries, bruises or wounds to the oral tissues. In this case, urgent safety measures will be taken promptly by the team to ensure the safety and protection of the participants’ health. Patients may stop the trial if considered necessary (or they may continue if the Institutional Review Board consider that it is safe once the problems have resolved)

### Dissemination of results

At the end of the study, all the data will be skimmed and an article will be written and submitted to the principal orthodontics journals. Additionally, the results will be communicated to participants, healthcare professionals and the general public. Communication to participants will be via email or letter. Communication to healthcare professionals will be via journals, meetings, congresses, and conferences.

## Additional file

Additional file 1: SPIRIT 2013 Checklist: recommended items to address in a clinical trial protocol and related documents. (PDF 3047 kb)

## Abbreviations

CBCT: Cone-beam computed tomography
CRF: Case Report Form
DICOM: Digital Imaging and Communication in Medicine
FOV: Field of view
GIPL: Guys Image Processing Lab
IBGE: Instituto Brasileiro de Geografia e Estatística
ITT: Intention-to-treat
STL: StereoLithography
UERJ: Universidade do Estado do Rio de Janeiro
WRL: Virtual Reality World
